# The core genes of cuproptosis assists in discerning prognostic and immunological traits of clear cell renal cell carcinoma

**DOI:** 10.3389/fonc.2022.925411

**Published:** 2022-09-21

**Authors:** Binxiang Chu, Zhenghua Hong, Xiaohe Zheng

**Affiliations:** ^1^Departmentof Orthopedic, Taizhou Hospital of Zhejiang Province Affiliated to Wenzhou Medical University, Linhai, China; ^2^Department of Pathology, Taizhou Hospital of Zhejiang Province Affiliated to Wenzhou Medical University, Linhai, China

**Keywords:** cuproptosis, copper, clear cell renal cell carcinoma, tumor immune microenvironment, immunotherapy, prognosis

## Abstract

**Objective:**

Cuproptosis, a nascent and unique pattern of cell death, is poised to spark a new rush of biological research. Yet, the subsumed mechanism of cuproptosis in carcinoma is not wholly clarified. The exclusive aim of this work is to define a novel classification algorithm and risk-prognosis scoring framework based on the expression modalities of cuproptosis genes to monitor clear cell renal cell carcinoma (ccRCC) patients’ prognosis and immunotherapeutic response.

**Methods:**

We pooled ccRCC data from three large-scale databases as the training subset and gathered a panel of clinical queues, termed the Taizhou cohort, which served as the validation setup. Wilcox test was conducted for comparison of expression variation, while the cox analysis and KM curves were utilized to visualize prognosis. Unsupervised clustering analysis was used to identify cuproptosis phenotypes in ccRCC. Concurrently, LASSO regression-based computational scoring model. A step further, gene set enrichment analysis (GSEA) was performed to check potential biological processes and the “CIBERSORT” R package was used to estimate the proportion of immune cells. To last, immunohistochemistry and qRT-PCR were carried out for the assay of critical genes for cuproptosis.

**Results:**

Here, we glimpse the prognostic power of cuproptosis genes in pan-cancer by investigating 33 cancers with multi-omics data to map their genetic heterogeneity landscape. In parallel, we devoted extra attention to their strategic potential role in ccRCC, identifying two phenotypes of cuproptosis with different immune microenvironmental characteristics by pooling ccRCC data from three large-scale databases. Additionally, we compiled a cuproptosis scoring system for clinicians to determine the prognosis, immunotherapy response, and chemosensitivity of ccRCC patients. Notably, we assembled a clinical cohort sample to validate the pivotal gene for cuproptosis, FDX1, to supply more clues to translate the biological significance of cuproptosis in ccRCC.

**Conclusion:**

In all, our investigations highlight that cuproptosis is involved in various components of ccRCC and assists in the formation of the tumor immune microenvironment. These results provide partial insights to further comprehend the molecular mechanisms of cuproptosis in ccRCC and could be helpful for the development of personalized therapeutic strategies targeting copper or cuproptosis.

## Introduction

The prevalence of clear cell renal cell carcinoma (ccRCC) persists highly worldwide (1). Epidemiological statistics show that well over 300,000 individuals have suffered from RCC worldwide, and 70% of those pathological types are ccRCC (2). Unlike early ccRCC, which could be satisfactorily surgically manipulated, metastatic ccRCC tends to exhibit resistance to conventional chemotherapy (3). As per the statistics, the 5-year survival rate for RCC is about 70%, while for those with late-stage, the rate is dismally low at 11.7% (4). Materially valid pyrrhic biological markers to inform treatment decisions or assess prognosis remain sorely lacking.

The effectiveness of trials of targeted regimens for ccRCC had revolutionized the treatment options for this disease-particularly the application of targeted vascular endothelial growth factor (VEGF) and immune checkpoint inhibitors (ICI) (5). A pioneering clinical trial of combination VEGF and ICI has yielded statistically significant beneficiaries in certain cases, opening up fresh horizons for the management of end-stage ccRCC (6). Over the last decade, rapid progress has been recorded in the treatment of nephrogenic tumors, especially in the area of both immunotherapy and targeted therapies (7). Notwithstanding, the available targeted and combination therapies have failed to have a powerful impact on disease progression and survival (8). The emergence of drug resistance forcing us to rethink tactics for the care of ccRCC. Undoubtedly, current investigations point to the notion that the response of ccRCC patients to either immunotherapy or chemotherapy is modulated by the tumor immune microenvironment (9). ccRCC, an immunogenic neoplasm, is well known to have a strong capacity to mediate immune dysfunction by provoking infiltration of immunosuppressive cells, as well as regulatory T cells, into the tumor microenvironment (TME) (10). It seems to be a feasible direction of investigation to formulate a line of systemic therapeutic regimens targeting the TME. Overall, the ultimate prognosis of ccRCC patients is not favorable. Therefore exploring new biomarkers and their intrinsic specific biological properties to identify poor prognosis and provide therapeutic targets is a tangible approach.

We note that the biological and therapeutic response of malignant cells is hampered by numerous modes of regulated cell death (RCD), ranging from apoptosis, which is the most renowned, to ferroptosis, the latest craze (11, 12). Induction of non-apoptosis-regulated cell death (RCD) has emerged as a neocon strategy to combat cancer, owing to the predominantly apoptotic nature of evil cells that have evolved to reject apoptosis. Moreover, a preponderance of data reveals crosstalk between RCD and anti-tumor immunity (13). Concretely, ferroptosis exhibits a synergistic anti-neoplastic immune response, while potentially suppressing the antitumor immune response (14). Thus, inducers or inhibitors targeting RCD might exert a more robust antitumor benefit when combined with immunotherapy applications, even in ICI-resistant individuals (15).

Metal ion homeostasis is crucial for cell survivorship, and heightened concentrations of certain metal ions could be harnessed to initiate various forms of RCD (16). Hence tremendous research is underway on the application or combination of metal ion vehicles for anti-neoplastic therapy. Copper (Cu) is not only an essential mineral nutrient for living organisms, but it also serves an integral role in a wide array of biological processes, ranging from mitochondrial respiration to inflammation and oxidative stress (17). Cu disposal disturbances show tremendous differences in outcome, from enhanced death phenotypes to rescued cells, which underscores the immense impact of the regulation of Cu homeostasis in mitochondria on normocytic physiology (18). When compared with healthy tissues, the Cu content is relatively increased in certain malignant cases, such as breast cancer, pancreatic cancer, prostate cancer, etc. (19–21). Besides, Cu accumulation is linked to enhanced cell proliferation, angiogenesis, and metastasis (22). It is well established that Cu homeostatic imbalance makes a substantial contribution to carcinogenesis, although scientists are debating whether it is a cause or a consequence of tumorigenesis (23). The remarkable progress in this field is evidenced by the uncovering of many other pipelines involving Cu and Cu-dependent molecules. Tsvetkov P et al. depicted a novel form of the Cu-elesclomol-triggered, mitochondrial tricarboxylic acid (TCA) dependent cell death denominated cuproptosis, which occurs by a mechanism independent of well-known apoptosis and pyroptosis (24). In this context, physicochemical carriers that can selectively induce cuproptosis may well succeed in surmounting the limitations of traditional anticancer pharmaceuticals and inject new hope for oncological management (25). However, the available reports on the association of cuproptosis with cancer almost exclusively involve alterations in protein expression levels without genomic mutations or epigenetic features. Our comprehension of cuproptosis and cuproptosis-related genes in cancerous growths is limited.

For this study, which is carried out based on available surveys and theoretical contexts, the key insights are as follows. For one, to systematically understand cuproptosis-related genes and unravel their mystery in human cancers, we integrated multi-omics data from 33 cancer types to comprehensively present a landscape of expression, prognosis, single-nucleotide variation (SNV), copy number variations (CNV), and methylation of these genes. It is not challenging to spot that they are heterogeneously altered in various neoplasms. Secondly, we are obsessed with the latent merits of cuproptosis in ccRCC, especially its intrinsic interlink with the immune system, and carry out clinical validation of the key gene FDX1 (26). From 3 independent databases, we merged genomic information of ccRCC samples in an attempt to disentangle the presumable role of cuproptosis genes in ccRCC. We were excited to detect a significant impact of cuproptosis on the tumor microenvironment, which was not yet reported in previous investigations. Moreover, We constructed a new risk-prognosis model, called CuproScroe, to facilitate the easy assessment of patient prognosis and therapeutic response. More in-depth, the above database-derived categorization and scoring phantoms were practice-tested in our local cohort. Our study provides significant insight into the function of cuproptosis in cancer.

## Materials and methods

### Collection of cuproptosis-related genes

Peter et al. proposed new insights into cuproptosis, from which we extracted 12 key genes, covering FDX1, LIAS, LIPT1, DLD, DLAT, PDHA1, PDHB, MTF1, GLS, CDKN2A, ATP7B, and SLC31A1, for the analysis of this study (24).

### Pan-cancer multi-omics analysis of cuproptosis-related genes

Gene expression data from 30 different tissues of normal subjects were collected from the GTEx dataset (V7.0) (https://commonfund.nih.gov/GTEx/) for gene expression analysis. Relevant mRNA sequence data, SNV data, CNV data, methylation data, and clinical data were captured from TCGA (https://portal.gdc.cancer.gov/) database for gene expression, SNV, CNV, methylation, and prognosis analysis for 33 cancers. Normalization of GTEx and TCGA mRNA data was carried out with reference to the multi-omics analysis strategy adopted by Jian Zhang et al. (27). CNV data were handled by GISTIC 2.0. Correlation analysis was run for each single gene to locate the methylation sites which corresponded most negatively to gene expression. The “maftools” R package was availed for SNV analysis.

### Cuproptosis phenotypes identification and CuproScroe model construction

In this investigation, we enrolled RNA sequencing data from the TCGA-KIRC cohort (containing 531 samples) from the TCGA database, the GSE29609 cohort (containing 39 samples) from the GEO database, and the E-MTAB-1980 cohort (containing 101 samples) from the ArrayExpress database. We merged data from 3 datasets and applied batch correction named the merge cohort. The “ComBat” algorithm of the “SVA” R package was adopted for batch correction of data sets. Unsupervised clustering analysis was used to identify cuproptosis phenotypes in ccRCC to categorize patients. “ConsensusClusterPlus” R package was designed to run each step.

Since cuproptosis genes did not show significant prognostic differences in the GEO database, we only integrated data from the TCGA and E-MTAB-1980 labeled the KIRC cohort. To be first, a cuproptosis score model, entitled CuproScore, was constructed using LASSO regression. The first step was to obtain the perfect model by the “cv.glmnet” system, initially screen the list of genes with zero β coefficient, and further confirm the optimal ones *via* multivariate Cox regression analysis. FDX1, LIAS, PDHB, and MTF1 opted for model configuration. Based on the linear combination of gene expression values and regression coefficients, risk scores were calculated for KIRC patients. The formula was as follow: Risk score=Σni Expi Coei (Exp = gene expression value; Coe = regression coefficient). Subsequently, univariate and multivariate Cox regression analyses were performed on the CuproScore model. The “rms” R package was on hand to create a nomogram.

### Enrichment and immune infiltration analysis

GO (Gene Ontology) and KEGG (Kyoto Encyclopedia of Genes and Genomes) enrichment analyses of cuproptosis genes were undertaken using “org.Hs.eg.db” and “clusterProfiler” R package. Meanwhile, gene set enrichment analysis (GSEA) was performed to check potential biological processes in high and low-risk groups. The “CIBERSORT” R package was used to estimate the proportion of immune cells.

### Drug sensitivity analysis

Via the TCIA database, immunophenotype scores (IPS) of KIRC sufferers were checked to pinpoint the part played by CuproScore in immunotherapy. The “pRRophetic” R package was selected to predict the drug sensitivity of four common chemotherapeutic agents for ccRCC therapy, including sorafenib, sunitinib, pazopanib, and axitinib. The methodology is based on a ridge regression algorithm to calculate the half-maximum inhibitory concentration (IC50).

### Histology hematoxylin-eosin (HE) and immunohistochemistry (IHC) staining

We retrospectively enrolled 142 patients who had radical nephrectomy or local mass resection at Taizhou Hospital from January 1, 2019, to March 24, 2022. Excluding some cases with imperfect clinicopathological information or missing follow-up, 127 cases were finally enlisted, which we named the Taizhou cohort. The study followed the guiding principles of the Declaration of Helsinki and was approved by the ethical review committee of Taizhou Hospital. Since we used discarded histological specimens, all waived written informed consent. Immunohistochemical staining for FDX1 (1:100, Abcam, ab108257) was carried out using paraffin sections of the tumor and normal kidney tissue adjacent to the tumor, as described in the method of reference (24). Image-Pro Plus (version 6.0) software was applied to evaluate the area and density of the stained regions, as well as the integrated optical density (IOD) values of the IHC slices. In addition, we performed the corresponding HE staining.

### Quantitative real-time polymerase chain reaction (qRT-PCR)

After paraffin tissue was dewaxed, RNA was isolated by Trizol reagent (Invitrogen, United States). Then, complementary DNA (cDNA) was synthesized *via* PrimeScriptTM RT kit (Takara). qRT-PCR analysis was run on the SYBR Premix Ex Taq (Takara). Normalization of all expression databases to GAPDH (as an endo-controller gene) using the 2−ΔΔCT method. Primer sequences referred to **Table S1**.

### Statistical analysis

R software (version 3.6.1) was used for all statistical analyses. Wilcoxon test was used for comparison between groups if not otherwise stated.

## Results

### Multi-omics genetic characterization and prognostic landscape of cuproptosis genes

Cuproptosis is a fresh conception, and the expression pattern and possible prognostic potential of genes responsible for cuproptosis in human carcinomas have intrigued us dramatically. Cuproptosis genes are differentially expressed in various human tissues, with FDX1 being significantly highly expressed in the adrenal gland (**Figure S1A**). Meanwhile, these genes were widely detected in the pan-cancer dataset. We noted that CDKN2A was significantly upregulated in CESC, while FDX1 was markedly downregulated in PCPG (**Figure S1B**). The mutation is an essential feature of tumors (28). As shown in **Figure S1D**, we estimated a total SNV frequency of 98.23% of cuproptosis genes in pan-cancer, among which the top gene in mutation frequency was CDKN2A (42%). The missense mutation was the primary mutation type. Missense mutations were the major mutation type and the mutation frequencies of SKCM, HNSC, LUSC and UCEC were more common. From the CNV pie chart (**Figure S1E**), we conclude that the main CNV types in cuproptosis genes were heterozygous amplification and deletion. However, the types of CNV in different tumors varied for different genes. For example, DLD predominantly exhibited heterozygous amplification, while LIAS was dominated by heterozygous deletion. Moreover, we noticed differential methylation alterations of genes in different tumor types (**Figure S1C**). Undoubtedly, these genes are highly heterogeneous genetic alterations in cancer, and we were not surprised to observe that they are remarkably associated with the prognosis of some neoplasms (**Figure S1F**). These results indicate that dysregulated expression of certain cuproptosis genes might be affiliated with tumorigenesis.

### Biological evaluation of cuproptosis genes in ccRCC

**Figure 1:**
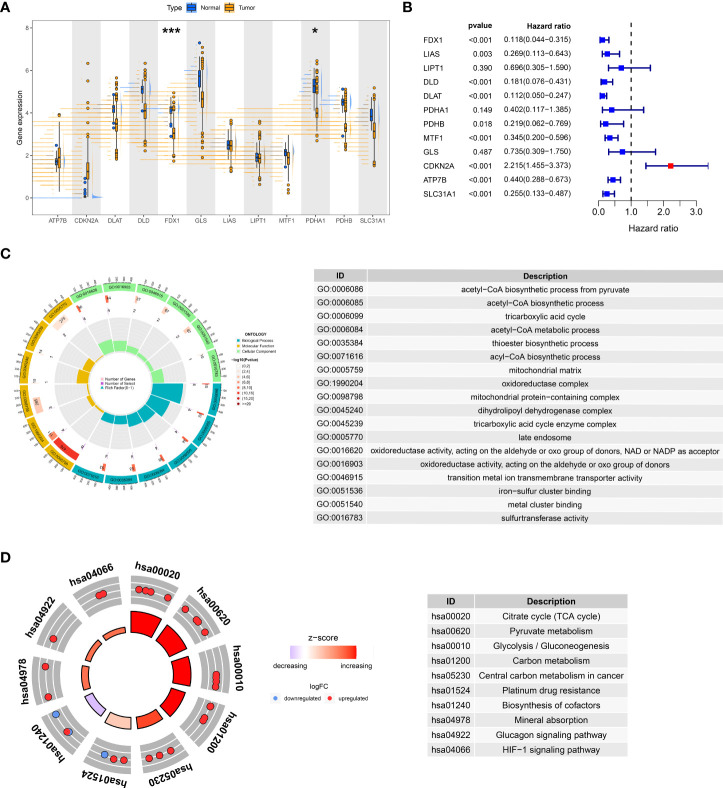
Expression and prognosis of cuproptosis genes in the TCGA-KIRC cohort. **(A)** Variable expression distribution of cuproptosis genes in TCGA-KIRC. **(B)** Cox regression analysis of TCGA-KIRC with cuproptosis genes. GO **(C)** and KEGG **(D)** analysis of cuproptosis genes. ^*^P< 0.05, ^***^P< 0.001.

We focused on the intrinsic mechanism of cuproptosis genes in ccRCC. In the TCGA-KIRC dataset, some genes were found to be differentially altered in normal and tumor tissues, especially FDX1 and PDHA (**Figure 1A**, *P*< 0.05). Cox regression analysis disclosed that FDX1, LIAS, DLD, DLAT, PDHB, MTF1, CDKN2A, ATP7B, and SLC31A1 were critical preventive factors for ccRCC, while CDKN2A acted as a risk factor (**Figure 1B**). The above positive results for expression and prognosis spark our curiosity about their biological functions. GO analysis hinted that these genes may be linked to the acetyl-CoA biosynthetic process from pyruvate, mitochondrial matrix, and oxidoreductase activity (**Figure 1C**). Furthermore, KEGG analysis highlighted the significant enrichment of these genes in the citrate (TCA) cycle, pyruvate metabolism, and glycolysis/gluconeogenesis (**Figure 1D**). All signs point to a possible tight link between cuproptosis and the pathogenesis of ccRCC.

### Two different cuproptosis phenotypes identified by unsupervised learning

**Figure 2:**
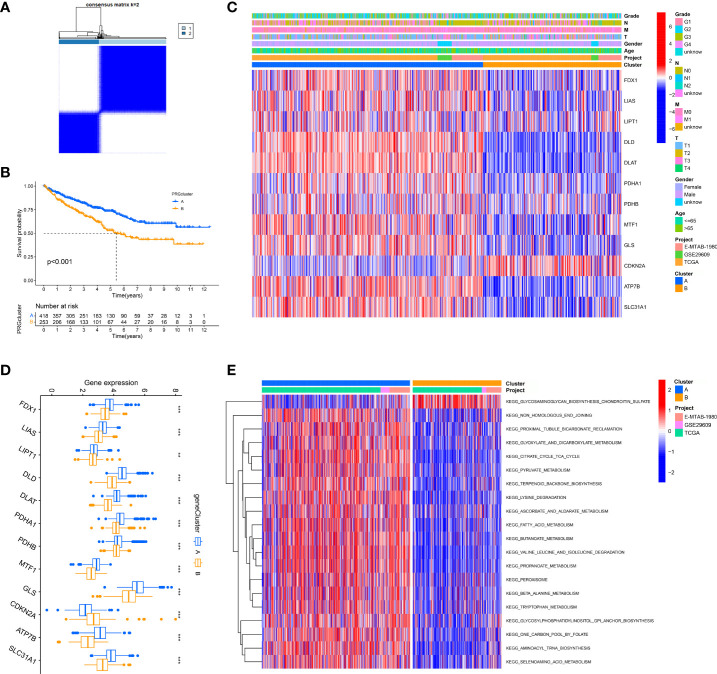
Unsupervised learning for cuproptosis classification. **(A)** Unsupervised clustering of 12 cuproptosis genes and the optimized consensus matrix with k = 2. **(B)** Kaplan-Meier Curves for differential survival of two cuproptosis phenotypes in the merge cohort which contains three datasets: TCGA, GEO, and E-MTAB-1980. **(C)** Heat map of clinical characteristics of two cuproptosis phenotypes. **(D)** Variable expression of cuproptosis genes in two phenotypes. **(E)** Heat map of the activation state of the top 20 KEGG pathways in different phenotypes. ^**^P< 0.01, ^***^P< 0.001.

**Figure 3:**
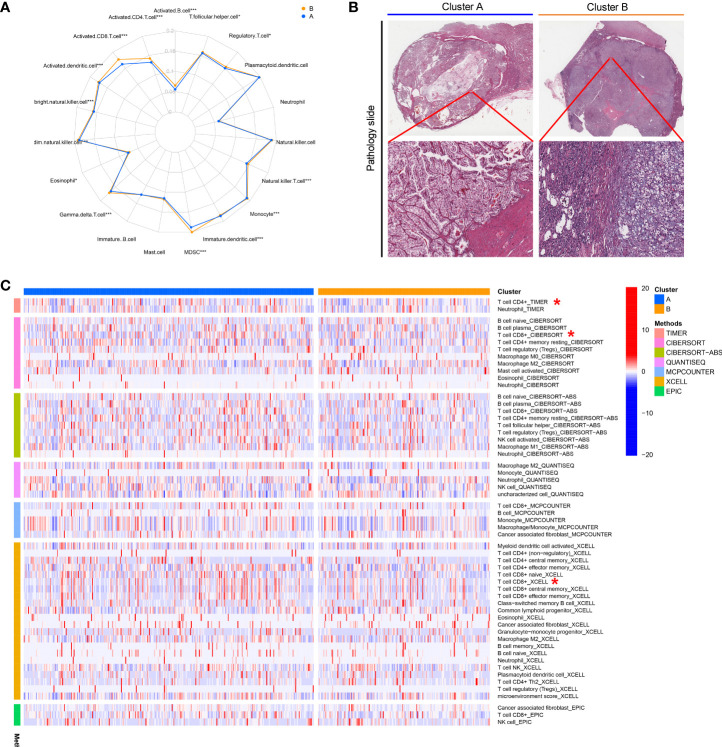
The immune landscape and biological characteristics of two cuproptosis phenotypes. **(A)** Radar chart of discrepancy immune infiltration of two phenotypes. **(B)** Representative images of the pathological HE staining of the two phenotypes from the TCGA dataset. **(C)** The enrichment levels of immune cells in two phenotypes. ^*^P< 0.05, ^***^P< 0.001.

We merged three dates of ccRCC in the public database, including TCGA-KIRC, GSE29609, and E-MTAB-1980, and classified them into two new phenotypes using an unsupervised algorithm, named A and B (**Figure 2A**). Kaplan-Meier analysis showed prognostic differences between the two phenotypes. Patients in B had the worse prognosis, while those in A had the better (**Figure 2B**; **S2**). There is a variable expression of cuproptosis genes in different phenotypes, with the majority of them being relatively highly expressed in A (**Figure 2D**). To further investigate the intrinsic biological differences between the different phenotypes to clarify differences in prognosis, we undertook a GSEA analysis. There were differences in pathway enrichment alterations between phenotypes, such as A mainly enriched in pyruvate metabolism, while B mainly enriched in glycosaminoglycan biosynthesis chondroitin sulfate (**Figure 2E**, and **Table S2**). It was essential to dig further into the TME differences in the two clusters since the TME was firmly tied to the prognosis and management of ccRCC. The analysis of TME cell infiltration revealed that significant variations in the level of immune cell infiltration between the two phenotypes (**Figures 3A, B**) and B were mainly enriched for infiltration of cellular immune cells, including CD4 T cell and CD8 T cell (**Figure 3C**, *P*< 0.05). In addition, we presented gene expression and clinicopathological features in different phenotypes with a heat map (**Figure 2C**). The above-mentioned results suggest a new classification of ccRCC, which could help distinguish prognosis and tumor immune status.

### Clinical significance of CuproScore

**Figure 4:**
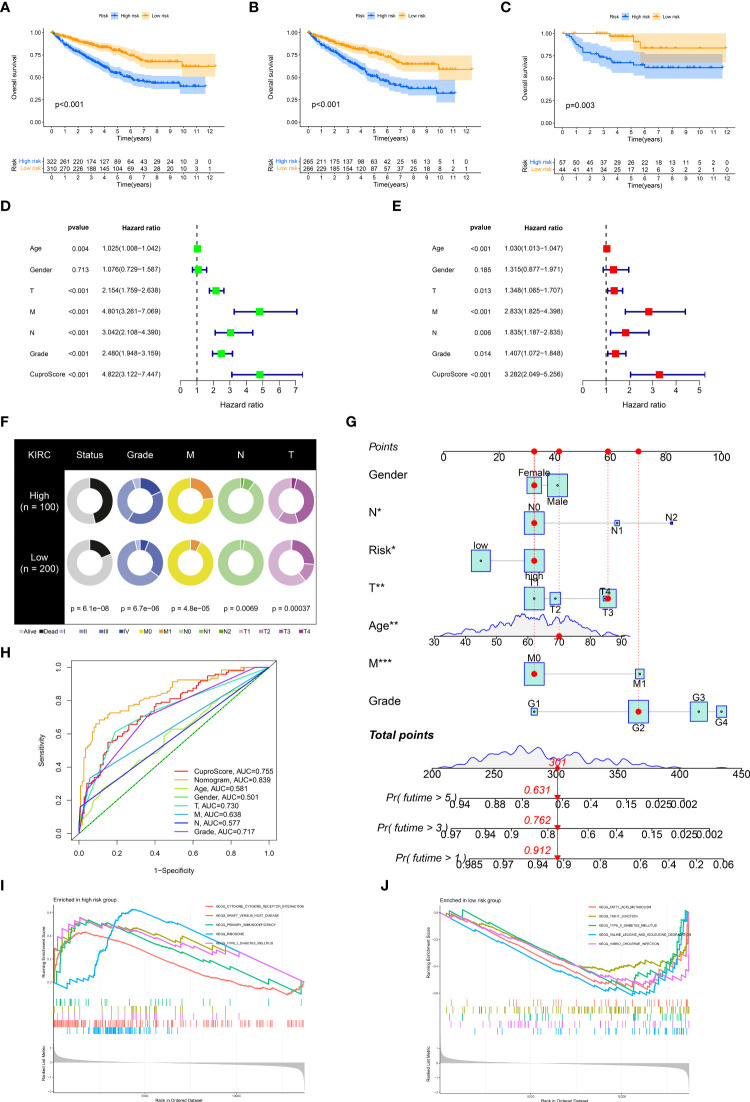
Clinical relevance and biological characteristics of CuproScore. Kaplan-Meier Curves for differential survival of two risk groups in the KIRC cohort **(A)**, TCGA **(B)**, and E-MTAB-1980 **(C)** cohort. Univariate **(D)** and multivariate **(E)** Cox regressions confirm CuproScore as an independent prognostic factor. **(F)** Clinical characteristics of the high and low scoring groups. **(G)** A nomogram for predicting the 1 year, 3 years, and 5 years survival rates of patients. **(H)** ROC curves for different risk factors. KEGG pathway enrichment analysis in the high-risk group **(I)** and low-risk group **(J)**. ^*^P< 0.05, ^**^P< 0.01, ^***^P< 0.001.

After confirming the prognostic significance and the critical role for the immune microenvironment of cuproptosis genes, we constructed a scoring model entitled CuproScore based on the 4 cuproptosis genes (which consist of FDX1, LIAS, PDHB, and MTF1) to measure the level of cuproptosis in ccRCC. Based on the median score, patients were ranked as high risk and low risk. By Kaplan-Meier analysis, we showed that the model could accurately predict prognosis, those with higher risk had a worse prognosis. This was confirmed in the KIRC cohort (**Figure 4A**), TCGA-KIRC (**Figure 4B**), and E-MTAB-1980 cohort (**Figure 4C**). In particular, through univariate (**Figure 4D**) and multivariate (**Figure 4E**) Cox analysis, we verified that the model could independently predict the prognosis of patients with ccRCC. Additively, univariate (**Figure S3A**) and multivariate (**Figure S3B**) outcomes signaled CuproScore as an independent prognostic measure for the E-MTAB-1980 cohort. Moreover, to facilitate clinician assessment, we also constructed nomo plots for presenting convenient quantitative methods applicable to predict patients’ 1-year, 3-year, and 5-year OS (**Figure 4G**) and confirmed the better prognostic, predictive validity of nomo plots by AUC curves (**Figure 4H**). Then, the donut chart summarizes the clinicopathological characteristics of the high- and low-risk groups (**Figure 4F**). To dig deeper, we aimed to use GSEA to explore the differences in intrinsic molecular mechanisms comparing the high- and low-risk groups and revealed that pathways associated with the high-risk group included cytokine receptor interaction, graft versus host disease, and primary immunodeficiency (**Figure 4I**), while pathways associated with the low-risk group included fatty acid metabolism, tight junction, and type II diabetes mellitus (**Figure 4J**).

### Characterization of the immune microenvironment in CuproScore

**Figure 5:**
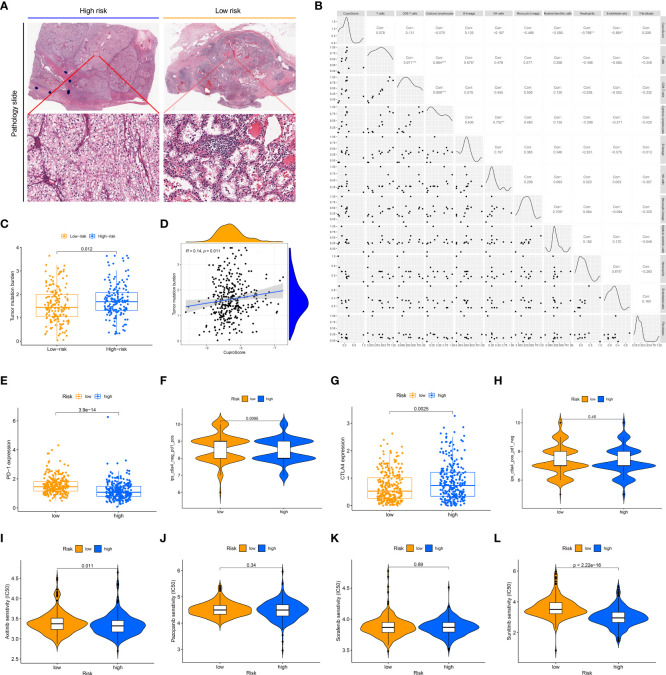
The immune landscape of the high and low CuproScore groups. **(A)** Representative images of the pathological HE staining of the high or low-risk group from the TCGA dataset. **(B)** Bubble chart of the correlation between CuproScore and immune cells. **(C)** Comparative tumor mutation burden in high and low-risk groups. **(D)** Correlation analysis of CuproScores and tumor mutation burden. Differential expression of PD-L1 **(E)** and relative odds of response to anti-PD-1/PD-L1 antibody **(F)** in high and low-risk groups. Differential expression of CTLA4 **(G)** and relative odds of response to CTLA4 monoclonal antibody **(H)** in high and low-risk groups. Drug sensitivity analysis between high and low-risk groups, including Axitinib **(I)**, Pazopanib **(J)**, Sorafenib **(K)**, and Sunitinib **(L)**. ^*^P< 0.05, ^**^P< 0.01, ^***^P< 0.001.

The tumor immune microenvironment is firmly linked to patient prognosis and immunotherapeutic response (29). We glimpsed that the low-risk group seemed to possess far more infiltration of immune cells (TCGA pathology slide, **Figure 5A**). To further explore the potential relationship between the CuproScore model and the immune system, we first performed an immune infiltration correlation analysis presenting the correlation between scores and immune cell infiltration. As shown in **Figure 5B**, the cuproScore was negatively correlated with neutrophils and endothelial cells-related signature scores. The present study establishes that tumor mutational burden (TMB) is closely tied to prognosis, with high TMB hinting at a worse prognosis (30). Our results pointed to a positive association between risk score and TMB (**Figure 5D**), with higher TMB in the high-risk group (**Figure 5C**), which partially accounts for the worse prognosis. All in all, the intrinsic connection of the cuproScore with the immune microenvironment and TMB may explain, to some extent, the differences in prognosis between the different risk groups.

### CuproScore to predict immunotherapy response and chemotherapy agents’ sensitivity

Immunotherapy brings new promise to ccRCC patients (31). However, only a minority of patients are receiving the benefits of immunotherapy, and there is an urgent need to develop new predictive systems to identify those who have a high response to immunotherapy to guide clinical precision treatment. In the CuproScore system we constructed, there was a variable expression of PD-L1 and CTLA4 in the high- and low-risk groups, with PD-1 being highly expressed in the low-risk group (**Figure 5E**) while CTLA4 being highly expressed in the high-risk group (**Figure 5G**). Importantly, our scoring system was capable of predicting anti-PD-1 treatment response, i.e., the low-risk group had a higher response (**Figure 5F**). It must be acknowledged that our scoring system was not perfect and there was no significant difference in treatment response to CTLA4 between the high and low-risk groups (**Figure 5H**). Chemotherapy is still the traditional therapeutic approach for ccRCC (32), despite the boom in immunotherapy. We evaluated the response of high- and low-risk groups to four clinically available chemotherapeutic agents (Axitinib, Pazopanib, Sorafenib, Sunitinib). A ridge regression algorithm was applied to the GDSC cell line dataset to assess the IC 50 values for each ccRCC sample, apart from Pazopanib (**Figure 5J**) and Sorafenib (**Figure 5K**), Axitinib (**Figure 5I**) and Sunitinib (**Figure 5L**) showed lower IC 50 values in the low-risk values, which suggests that the low-risk group may be more sensitive to these agents.

### Clinical validation of cuproptosis phenotypes and CuproScore in Taizhou cohort

**Figure 6:**
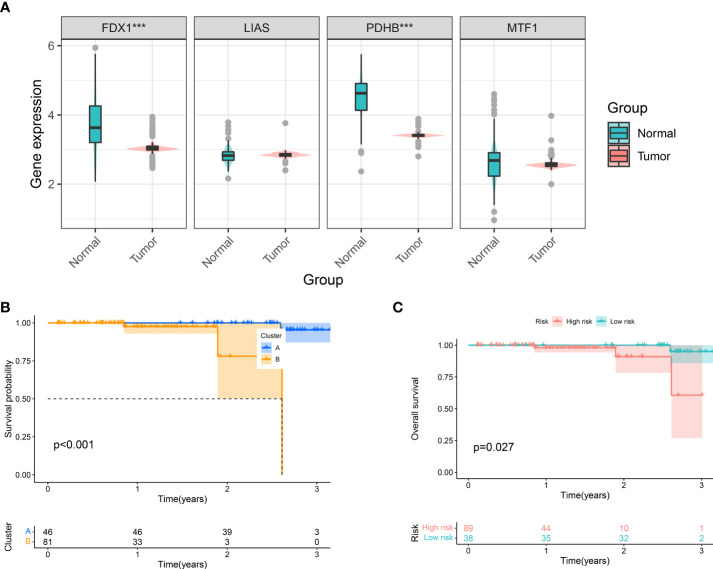
Clinical Validation of Cuproptosis Phenotypes and CuproScore. **(A)** Differential expression of FDX1, LIAS, PDHB, and MFT1 in Taizhou cohort. Kaplan-Meier Curves for differential survival of two cuproptosis phenotypes **(B)** and two risk groups **(C)** in Taizhou cohort. ***P< 0.001.

Via profiling of the data, we constructed cuproptosis phenotypes and a neoteric CuproSocre system with superior prognostic predictive capabilities. This called for more practical testing, so in the Taizhou cohort, we performed qRT-PCR experiments on 127 tumor tissues and their corresponding normal samples to obtain the expression of genes (FDX1, LIAS, PDHB, and MTF1). **Figure 6A** displays that FDX1 and PDHB were considerably downregulated in the tumors. Furthermore, we classified the Taizhou cohort into two clusters, A and B, based on gene expression patterns. Here, patients in cluster B had a worse prognosis (**Figure 6B**), which validated the clustering strategy of ccRCC. Based on the formula for Cuproscore model, we partitioned the Taizhou cohort into the high- and low-risk groups, and the results of KM analysis showed that those in the high-risk group suffer a worse prognosis (**Figure 6C**), which was consistent with the KIRC cohort from the datasets.

### Clinical validation of FDX1 in ccRCC

**Figure 7:**
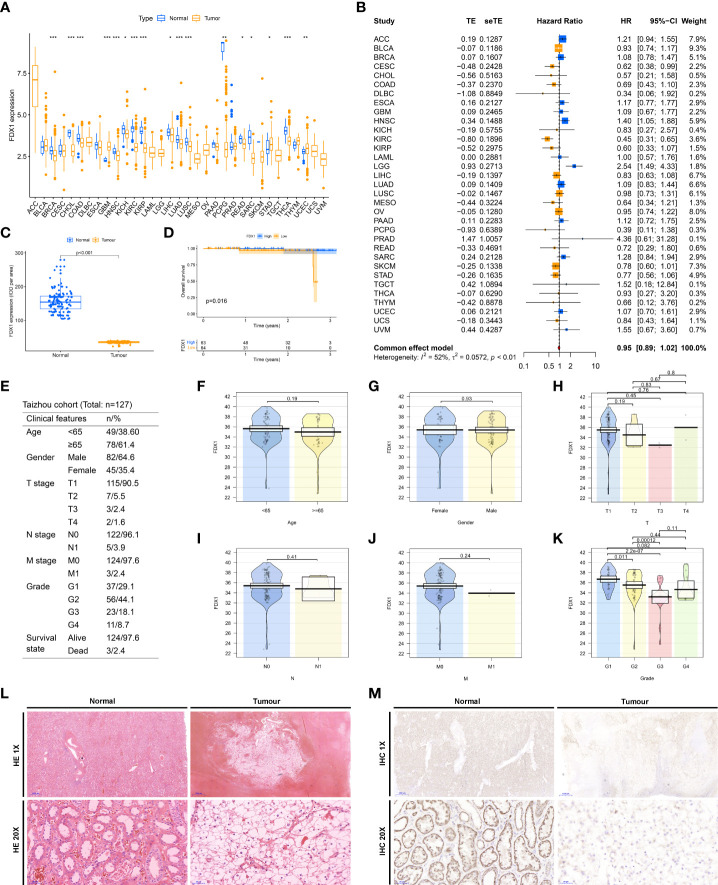
Clinical validation of FDX1. **(A)** Differential expression of FDX1 in TCGA pan-cancer. **(B)** A meta-analysis of the prognosis of pan-cancer with FDX1. **(C)** Differential expression of FDX1 in normal and tumor tissues in the Taizhou cohort. **(D)** Overall survival differences between high and low FDX1 expression groups in the Taizhou cohort. **(E)** Table of clinicopathological characteristics of the Taizhou cohort. Differences in clinicopathological characteristics, including Age **(F)**, and Gender **(G)**, T stage **(H)**, N stage **(I)**, and M stage **(J)**, Grade **(K)**, between high and low FDX1 expression groups in the Taizhou cohort. **(L)** HE staining of tumors and adjacent normal renal tissues in Taizhou cohort. **(M)** Immunohistochemical detection of FDX1 in tumors and adjacent normal renal tissues in Taizhou cohort. ^*^P< 0.05, ^**^P< 0.01, ^***^P< 0.001.

FDX1 is one of the pivotal genes for cuproptosis, and we are particularly intrigued by its hidden biological role in KIRC. By TCGA pan-cancer expression analysis, we observed that FDX1 was heterogeneously expressed in a multitude of neoplasms and markedly downregulated in tumor tissues of KIRC (**Figure 7A**). Meanwhile, a meta-analysis disclosed that FDX1 performed as a preventive factor in KIRC (**Figure 7B**). All trails of evidence tend to highlight the potential status of FDX1 in ccRCC as a novel prognostic factor. To further ascertain this exhilarating result, we gathered pathological specimens, complete clinicopathological data, and follow-up information from 127 ccRCC patients in our hospital. The expression of FDX1 in tumor and adjacent normal kidney tissue sections of all patients was detected by immunohistochemistry (**Figures 7L, M**, **S4**). In agreeance with the outcomes of database analysis, FDX1 was significantly hyper expressed in the normal kidney while weakly expressed in the tumor (**Figure 7C**). Patients were divided into high and low expression groups based on the median IOD values, and we showed that the low expression group had an unfavorable prognosis (**Figure 7D**). Notably, FDX1 expression only partially differed in grade (**Figure 7K**), while there were no differential alterations in age (**Figure 7F**), gender (**Figure 7G**), T stage (**Figure 7H**), N stage (**Figure 7I**), and M stage (**Figure 7J**). This might be concern with the small sample size enrolled in the Taizhou cohort. Eventually, **Figure 7E** and **Table S3** exhibit the clinicopathological features of our Taizhou cohort.

## Discussion

Cu impacts cell fate and is engaged in carcinogenesis (33). The newborn Cu-associated form of cell death, cuproptosis, is setting off a burning wave of research that could shed new light on oncotherapy (34). We focus on the critical role of cuproptosis in human cancers, especially ccRCC. In this work, we initially unveiled the multi-omic genetic landscape of cuproptosis genes in 33 neoplasms in humans, detecting their heterogeneous alterations and prognostic potential. Concretely, we clinically validated the pivotal gene for cuproptosis, FDX1. We polled ccRCC cohorts from 3 large tumor databases and identified two subtypes of cuproptosis in ccRCC. Furthermore, we constructed a new risk-prognosis model to facilitate the assessment of patient’s prognosis and response to therapies, labeled CuproScore, which is not as well able to delineate the difference in immune microenvironment status of patients, but also to discern patients’ response to partial immunotherapy and susceptibility to chemotherapeutic agents.

By locating that Cu levels in serum and tumor tissue are increased in cancer patients versus healthy individuals, combined with the knowledge that Cu might drive multiple modes of neoplastic cell death through a wide variety of mechanisms, including reactive oxygen species (ROS) accumulation, mitochondrial dysfunction, and anti-angiogenesis, researchers have demonstrated the participation of Cu in cancer therapies, such as chemokines, phototherapy, and chemotherapy (35, 36). Consequently, disruption of Cu homeostasis is an emerging strategy for anticancer therapy (37). A recent publication in Science revealed that intracellular Cu accumulation triggers the aggregation of mitochondrial lipid acylated proteins and destabilization of Fe-S cluster proteins, culminating in a unique type of cell death coined Cu toxicity (24). This breakthrough has ignited great enthusiasm for cuproptosis in the field of biology. The feeble expression of cuproptosis genes in certain cancers has been investigated, For instance, Goh W Q et al. observed that the DLAT was substantially upregulated in gastric cancer (38), while the FDX1 was decreased in lung adenocarcinoma (39), both of which are implicated in tumor progression by influencing tumor metabolic pathways. We also obtained confirmation of variable expression of cuproptosis genes in the tumor by pan-cancer analysis, hinting at a possible chance of targeting cuproptosis in tumors. Besides, epigenetic alterations are not only involved in tumorigenesis but also closely interlinked with tumor metastasis, recurrence, treatment, and prognosis (40, 41). We remarked extensive SNV, CNV, and methylation alterations of cuproptosis genes in carcinogenesis, which might help to interpret the genetic essence of cuproptosis affecting cancer prognosis in a multidimensional approach.

There is rising proof that cuproptosis is involved in innate and adaptive immune responses (42, 43), but its mechanism in reshaping the tumor microenvironment in ccRCC remains poorly understood. Following the expression profile of cuproptosis regulators, we characterized two cuproptosis-associated isoforms with different immune microenvironmental features. By calculating the correlation coefficient between immune infiltrating cells and different phenotypes, we figured out that B phenotypes with higher immune cell infiltration have a better prognosis, which is consistent with the classical findings that tumor-infiltrating CD8+ T cells are a sign of poor prognosis (44). Another contribution is that we created a scoring system to classify patients into high and low-risk groups based on cuproptosis-related genes. This scoring kit also correlates with tumor microenvironmental characterization of ccRCC and allows for a predictable prognosis.

ccRCC has a peculiar immunological profile in terms of pathogenesis and management that distinguishes it from other sorts of cancer that respond to ICI (10). Drug resistance is one of the crucial clinical burdens of ccRCC. The development of drug tolerance is down to numerous factors, ranging from impairment of apoptosis to infiltration of tumorigenic immune cells (45). Of late, new strategies have emerged as well, including immunotherapy, such as PD-1 inhibitors (Nivolumab) (46). Nonetheless, the overwhelming majority of patients respond minimally and treatment resistance is almost inevitable. In our CuproScore system, patients in the high-risk group featured a higher tumor mutational burden and less favorable response to anti-PD-1/PD-L1 therapy. This illustrates the power of the CuproScore to forecast the efficacy of PD-L1 immunotherapy. Likewise, in the case of traditional chemotherapy, it shows an extra capacity to predict patients’ sensitivity to partially medicine.

FDX1 is well known originally as an iron-sulfur protein participating in the synthesis of steroid hormones and the reduction of mitochondrial cytochromes (47, 48). More recently, investigators have revealed that it boosted elesclomol-induced Cu-dependent cell death and potentially offers fresh thoughts to heighten the efficacy of certain cancer-targeting drugs (24). It was noted in the latest articles that FDX1 is firmly associated with the metabolism of the three major nutrients (glucose, fatty acids, and amino acids) in lung adenocarcinoma and its down-regulated expression could be an indicator of poor prognosis (39). To our knowledge, the role of FDX1 on ccRCC has not been reported yet. It is the first time we found that it was statistically significantly down-regulated in ccRCC, with a pronounced impact on patient survival, as evidenced both by the results of genomic analysis of the database and by our clinical cohort.

We must admit that the present study contains certain limitations. Primarily, the validation cohort of this report is a single-center retrospective study and still awaits further validation by multi-center clinical cohort support. Despite the implementation of several immunohistochemical experiments, our findings imply that FDX1 may play an essential role in ccRCC, but more in-depth mechanisms lack exploration. Our team is handling further diligent work on this topic.

## Conclusion

**Figure 8:**
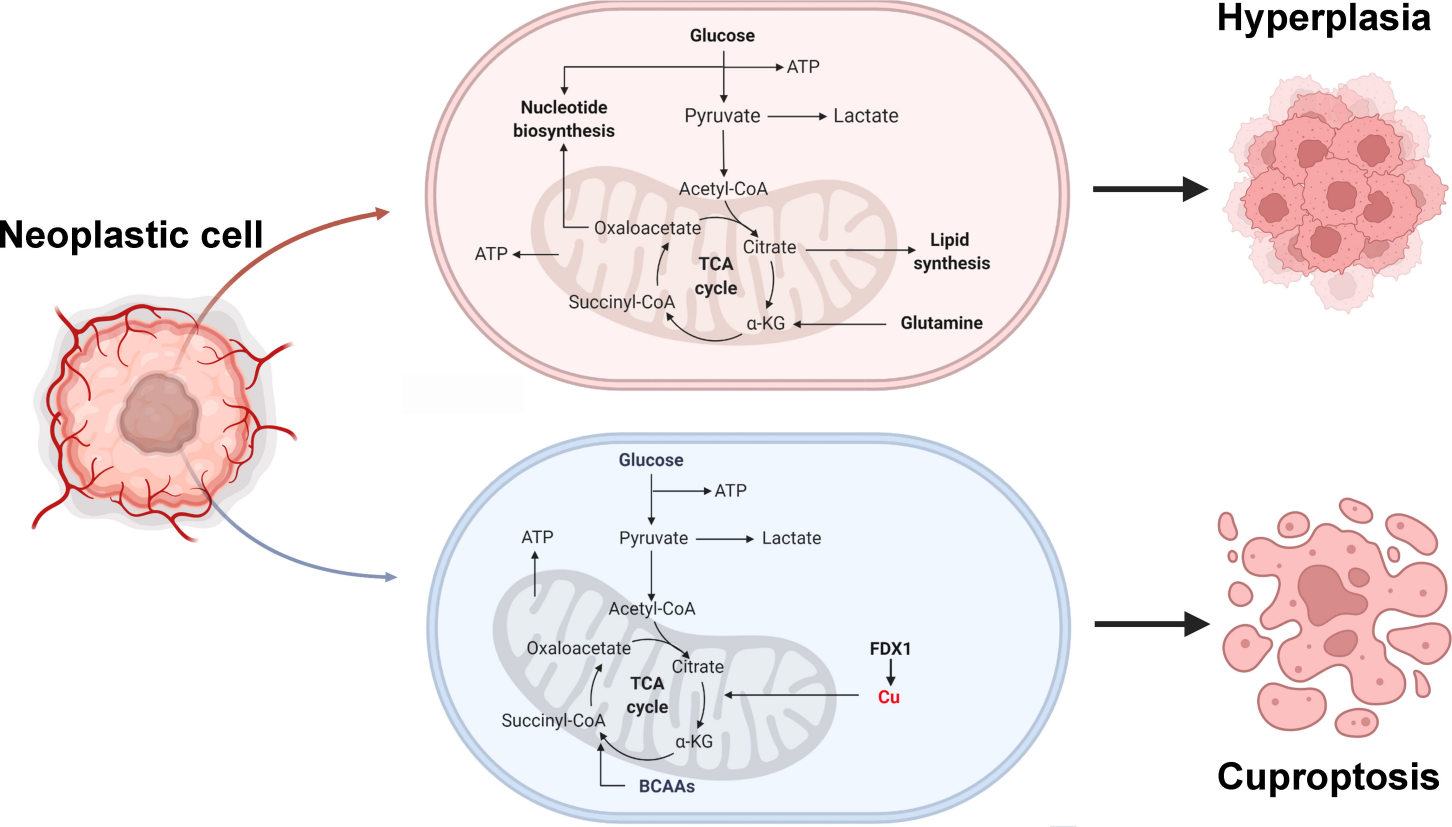
Tumor metabolism and cuproptosis mechanism. BCAAs: Branched-chain amino acids.

In outline, we comprehensively present a multi-omic landscape of the transcriptome, genome, and epigenome of cuproptosis genes in multiple human cancers, partially explaining the intrinsic mechanisms of cuproptosis genes carcinogenesis (**Figure 8**). Furthermore, we identified a new classification of ccRCC based on cuproptosis genes expression-based features and constructed a new risk-prognosis model, called CuproScore. These new conclusions, clinical classifications, and models will help provide fresh insight into the overall understanding of the role of cuproptosis in cancer and the development of new therapeutic targets for tumors. Nevertheless, these results still require further experiments and large-scale clinical practice.

## Data availability statement

The raw data supporting the conclusions of this article will be made available by the authors, without undue reservation.

## Ethics statement

The studies involving human participants were reviewed and approved by the ethical review committee of Taizhou Hospital. The patients/participants provided their written informed consent to participate in this study. Written informed consent was obtained from the individual(s) for the publication of any potentially identifiable images or data included in this article.

## Author contributions

BC, ZH, and XZ participated in designing and writing the manuscript. All authors contributed to the article and approved the submitted version.

## Funding

This work was sponsored by the National Natural Science Foundation of China (80212076) and Medical Health Science and Technology Project of Zhejiang Provincial Health Commission (2020384729).

## Conflict of interest

The authors declare that the research was conducted in the absence of any commercial or financial relationships that could be construed as a potential conflict of interest.

## Publisher’s note

All claims expressed in this article are solely those of the authors and do not necessarily represent those of their affiliated organizations, or those of the publisher, the editors and the reviewers. Any product that may be evaluated in this article, or claim that may be made by its manufacturer, is not guaranteed or endorsed by the publisher.

## Supplementary material

Supplementary Figure 1 Pan-cancer multi-omics expression and prognostic schema of cuproptosis genes. **(A)** The expression value of cuproptosis genes in the GTEx dataset. **(B)** Expression of cuproptosis genes in TCGA dataset. **(C)** Bubble chart of differences in the methylation of cuproptosis genes. **(D)** Waterfall diagram of somatic mutations in the 10 top cuproptosis genes with the most frequent mutations in pan-cancer. **(E)** CNV percentage of cuproptosis genes in each cancer. **(F)** Cox regression analysis of pan-cancer with cuproptosis genes.

Supplementary Figure 2 Kaplan-Meier Curves for differential survival of two cuproptosis phenotypes in the merge cohort which contains two datasets: TCGA and E-MTAB-1980.

Supplementary Figure 3 The application merit of CuproScore in the E-MTAB-1980 cohort. Univariate **(A)** and multivariate **(B)** Cox regressions confirm CuproScore as an independent prognostic factor in the E-MTAB-1980 cohort.

Supplementary Figure 4 Immunohistochemical detection of FDX1 in tumors of different grades in Taizhou cohort.
