# Supplementary figures and images for: The core genes of cuproptosis assists in discerning prognostic and immunological traits of clear cell renal cell carcinoma
Source: Front Oncol. 2022 Sep 21;12:925411. doi: 10.3389/fonc.2022.925411 (PMC9533068; doi:10.3389/fonc.2022.925411)

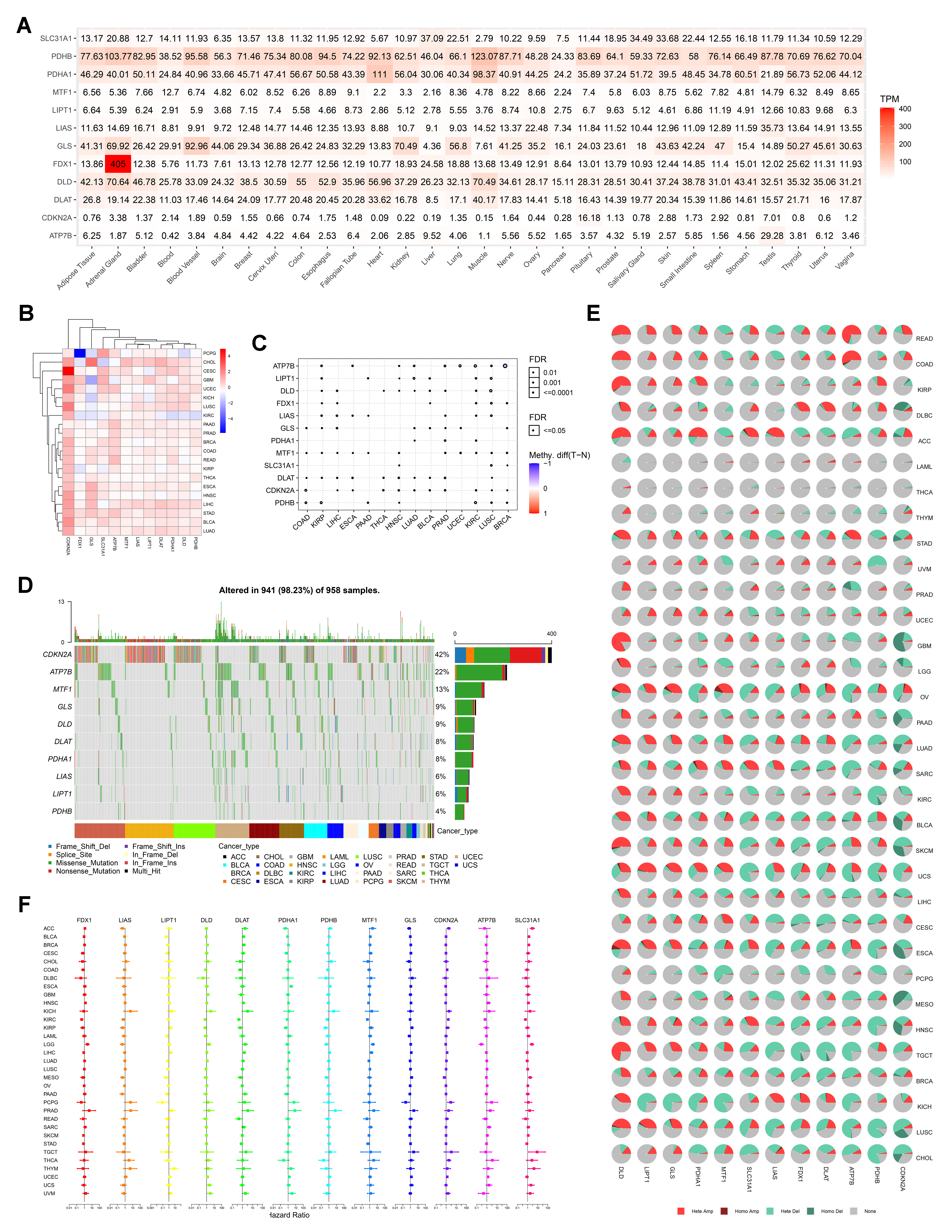

Supplement: Supplementary Figure 1 — Pan-cancer multi-omics expression and prognostic schema of cuproptosis genes. (A) The expression value of cuproptosis genes in the GTEx dataset. (B) Expression of cuproptosis genes in TCGA dataset. (C) Bubble chart of differences in the methylation of cuproptosis genes. (D) Waterfall diagram of somatic mutations in the 10 top cuproptosis genes with the most frequent mutations in pan-cancer. (E) CNV percentage of cuproptosis genes in each cancer. (F) Cox regression analysis of pan-cancer with cuproptosis genes. [file Image_1.tif]

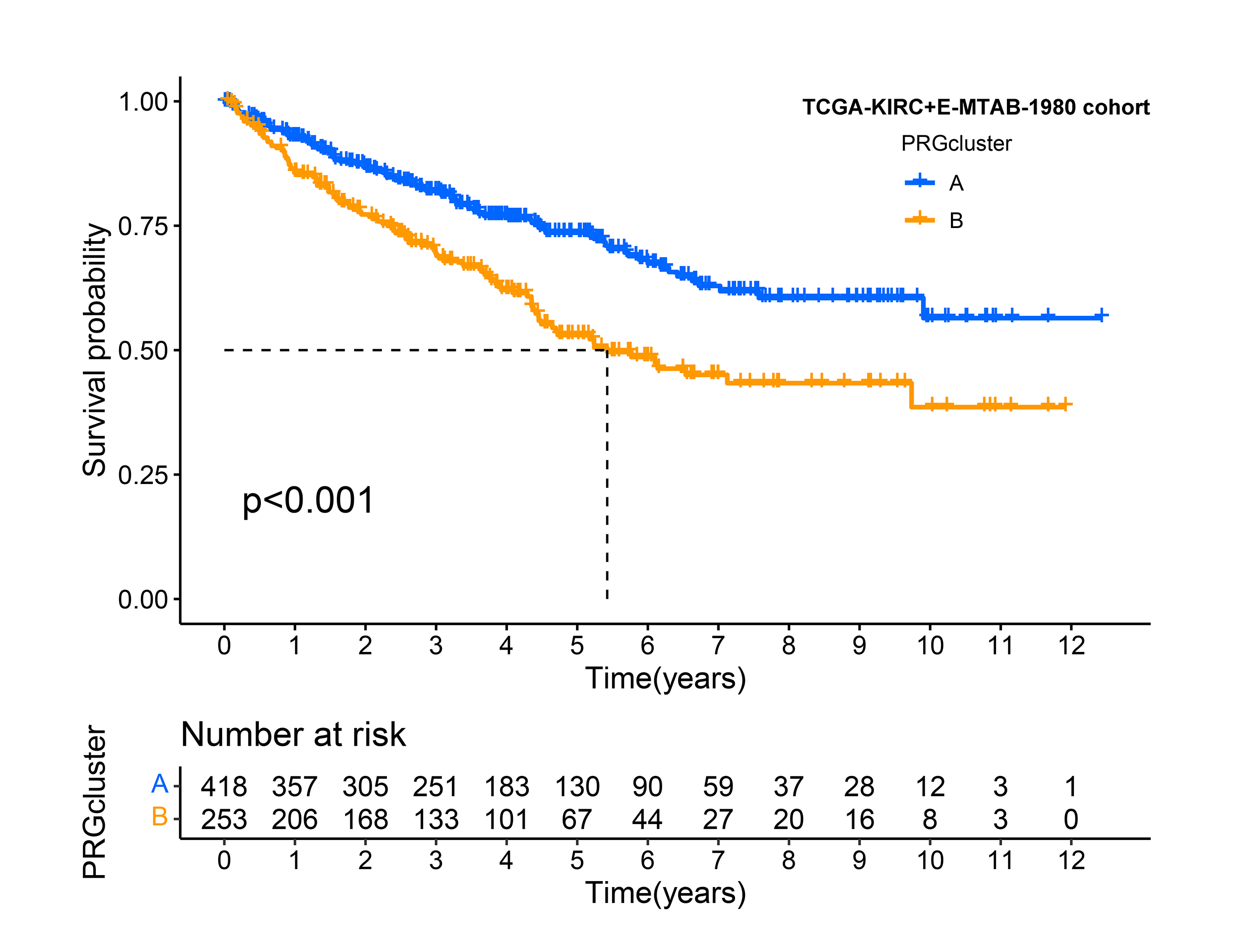

Supplement: Supplementary Figure 2 — Kaplan-Meier Curves for differential survival of two cuproptosis phenotypes in the merge cohort which contains two datasets: TCGA and E-MTAB-1980. [file Image_2.tif]

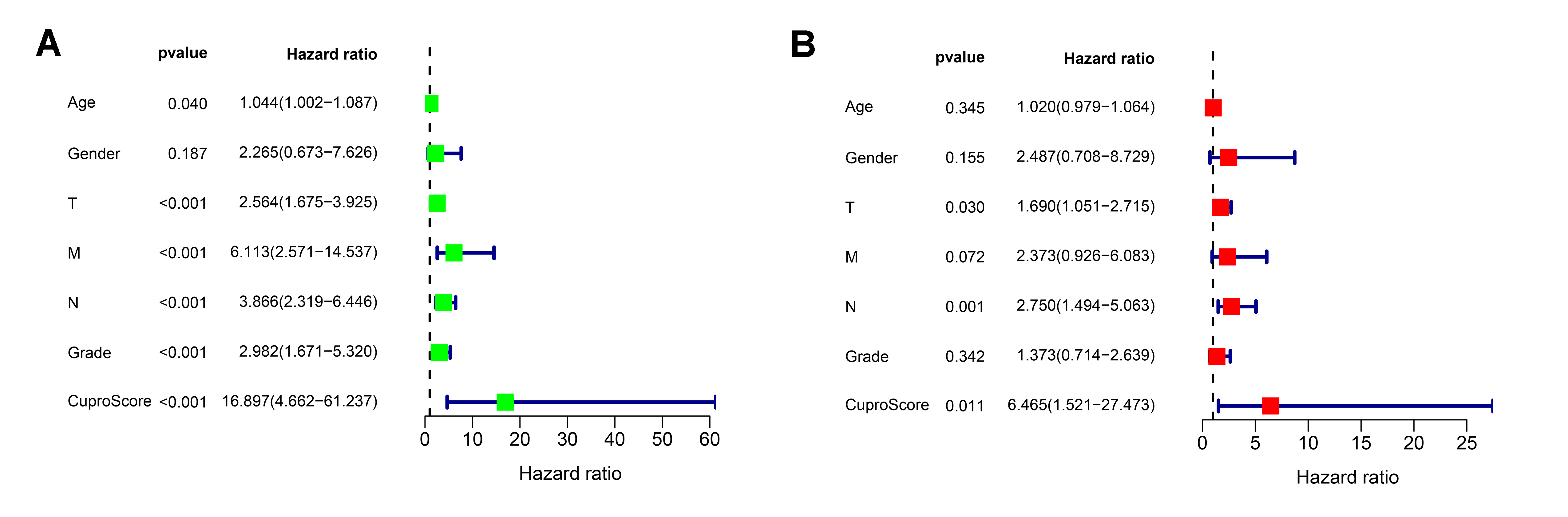

Supplement: Supplementary Figure 3 — The application merit of CuproScore in the E-MTAB-1980 cohort. Univariate (A) and multivariate (B) Cox regressions confirm CuproScore as an independent prognostic factor in the E-MTAB-1980 cohort. [file Image_3.tif]

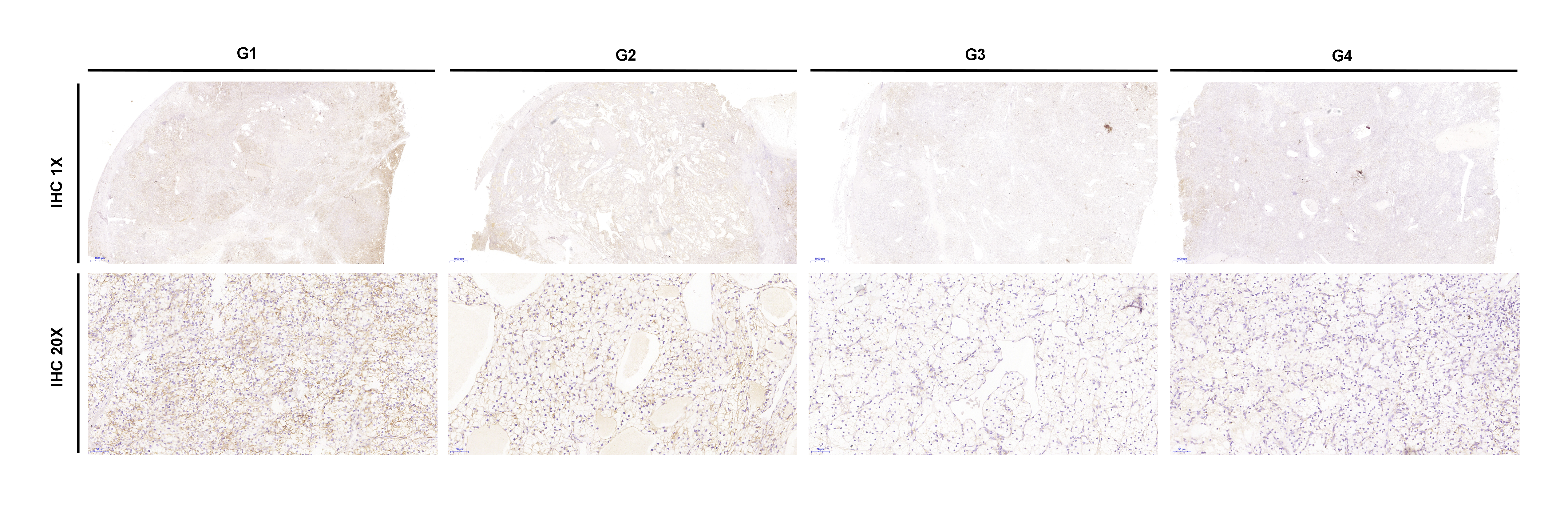

Supplement: Supplementary Figure 4 — Immunohistochemical detection of FDX1 in tumors of different grades in Taizhou cohort. [file Image_4.tif]
