# Supplementary material for: The core genes of cuproptosis assists in discerning prognostic and immunological traits of clear cell renal cell carcinoma
Source: Front Oncol. 2022 Sep 21;12:925411. doi: 10.3389/fonc.2022.925411 (PMC9533068; doi:10.3389/fonc.2022.925411)
Supplement: Supplementary file 5 [file Table_1.docx]

**Table S1.** Primer sequence of qRT-PCR

| Gene | Sequence (5'->3') |
| --- | --- |
| FDX_Forward | CCTCTTTGGAGTCTCTCGCGG |
| FDX1_Reverse | CAGCCCAACCGTGATCTGT |
| LIAS_Forward | CCGTTAAGCTCCTTGCCAGA |
| LIAS_Reverse | GCCATAGAGGTTGAGGCTGG |
| PDHB_Forward | GAAGAGGCGCTTTCACTGGA |
| PDHB_Reverse | AGTGACACGAACAGCAGGAG |
| MTF1_Forward | GCCGCGGAGACAAGTCATTA |
| MTF1_Reverse | CAGCCATTACTGGGGCAGAA |
| GAPDH_Forward | GGAGCGAGATCCCTCCAAAAT |
| GAPDH_Reverse | GGCTGTTGTCATACTTCTCATGG |
