# Supplementary material for: The core genes of cuproptosis assists in discerning prognostic and immunological traits of clear cell renal cell carcinoma
Source: Front Oncol. 2022 Sep 21;12:925411. doi: 10.3389/fonc.2022.925411 (PMC9533068; doi:10.3389/fonc.2022.925411)
Supplement: Supplementary file 6 [file Table_2.docx]

**Table S2.** All differential signaling pathways between Cluster A and B

| Description | adj.P.Val |
| --- | --- |
| KEGG_VALINE_LEUCINE_AND_ISOLEUCINE_DEGRADATION | 1.81E-69 |
| KEGG_PROPANOATE_METABOLISM | 6.62E-64 |
| KEGG_CITRATE_CYCLE_TCA_CYCLE | 6.62E-64 |
| KEGG_LYSINE_DEGRADATION | 3.72E-58 |
| KEGG_AMINOACYL_TRNA_BIOSYNTHESIS | 1.38E-57 |
| KEGG_FATTY_ACID_METABOLISM | 1.30E-44 |
| KEGG_ONE_CARBON_POOL_BY_FOLATE | 1.45E-43 |
| KEGG_BUTANOATE_METABOLISM | 9.44E-43 |
| KEGG_TERPENOID_BACKBONE_BIOSYNTHESIS | 1.58E-41 |
| KEGG_SELENOAMINO_ACID_METABOLISM | 8.34E-38 |
| KEGG_GLYCOSAMINOGLYCAN_BIOSYNTHESIS_CHONDROITIN_SULFATE | 5.05E-37 |
| KEGG_PYRUVATE_METABOLISM | 1.05E-36 |
| KEGG_GLYOXYLATE_AND_DICARBOXYLATE_METABOLISM | 1.53E-31 |
| KEGG_PROXIMAL_TUBULE_BICARBONATE_RECLAMATION | 5.91E-31 |
| KEGG_ASCORBATE_AND_ALDARATE_METABOLISM | 1.46E-29 |
| KEGG_NON_HOMOLOGOUS_END_JOINING | 4.88E-29 |
| KEGG_BETA_ALANINE_METABOLISM | 9.71E-29 |
| KEGG_TRYPTOPHAN_METABOLISM | 6.59E-28 |
| KEGG_PEROXISOME | 1.43E-27 |
| KEGG_GLYCOSYLPHOSPHATIDYLINOSITOL_GPI_ANCHOR_BIOSYNTHESIS | 1.23E-26 |
| KEGG_NEUROACTIVE_LIGAND_RECEPTOR_INTERACTION | 4.10E-26 |
| KEGG_INSULIN_SIGNALING_PATHWAY | 4.72E-26 |
| KEGG_ADIPOCYTOKINE_SIGNALING_PATHWAY | 8.13E-25 |
| KEGG_ENDOMETRIAL_CANCER | 8.74E-25 |
| KEGG_MTOR_SIGNALING_PATHWAY | 9.62E-25 |
| KEGG_TASTE_TRANSDUCTION | 4.32E-23 |
| KEGG_GLYCOLYSIS_GLUCONEOGENESIS | 1.29E-21 |
| KEGG_ALANINE_ASPARTATE_AND_GLUTAMATE_METABOLISM | 2.57E-21 |
| KEGG_UBIQUITIN_MEDIATED_PROTEOLYSIS | 4.06E-21 |
| KEGG_PENTOSE_AND_GLUCURONATE_INTERCONVERSIONS | 6.99E-21 |
| KEGG_NICOTINATE_AND_NICOTINAMIDE_METABOLISM | 2.74E-20 |
| KEGG_BIOSYNTHESIS_OF_UNSATURATED_FATTY_ACIDS | 3.34E-19 |
| KEGG_EPITHELIAL_CELL_SIGNALING_IN_HELICOBACTER_PYLORI_INFECTION | 4.52E-19 |
| KEGG_REGULATION_OF_AUTOPHAGY | 9.97E-19 |
| KEGG_COMPLEMENT_AND_COAGULATION_CASCADES | 1.57E-18 |
| KEGG_ERBB_SIGNALING_PATHWAY | 2.33E-18 |
| KEGG_PPAR_SIGNALING_PATHWAY | 2.92E-18 |
| KEGG_CYTOKINE_CYTOKINE_RECEPTOR_INTERACTION | 2.95E-18 |
| KEGG_ENDOCYTOSIS | 3.90E-18 |
| KEGG_NON_SMALL_CELL_LUNG_CANCER | 5.51E-18 |
| KEGG_HISTIDINE_METABOLISM | 1.10E-17 |
| KEGG_SPHINGOLIPID_METABOLISM | 1.28E-17 |
| KEGG_ARGININE_AND_PROLINE_METABOLISM | 4.38E-17 |
| KEGG_GLYCOSAMINOGLYCAN_BIOSYNTHESIS_KERATAN_SULFATE | 4.69E-17 |
| KEGG_RIBOSOME | 7.83E-17 |
| KEGG_AMYOTROPHIC_LATERAL_SCLEROSIS_ALS | 8.89E-17 |
| KEGG_OLFACTORY_TRANSDUCTION | 3.02E-16 |
| KEGG_PROSTATE_CANCER | 4.89E-16 |
| KEGG_DILATED_CARDIOMYOPATHY | 1.63E-15 |
| KEGG_RENIN_ANGIOTENSIN_SYSTEM | 7.51E-15 |
| KEGG_INOSITOL_PHOSPHATE_METABOLISM | 7.51E-15 |
| KEGG_PANCREATIC_CANCER | 1.13E-14 |
| KEGG_GLYCINE_SERINE_AND_THREONINE_METABOLISM | 6.84E-14 |
| KEGG_HYPERTROPHIC_CARDIOMYOPATHY_HCM | 9.32E-14 |
| KEGG_CYSTEINE_AND_METHIONINE_METABOLISM | 9.58E-14 |
| KEGG_ADHERENS_JUNCTION | 1.17E-13 |
| KEGG_HEMATOPOIETIC_CELL_LINEAGE | 1.22E-13 |
| KEGG_VASOPRESSIN_REGULATED_WATER_REABSORPTION | 1.53E-13 |
| KEGG_RENAL_CELL_CARCINOMA | 3.79E-13 |
| KEGG_PANTOTHENATE_AND_COA_BIOSYNTHESIS | 6.09E-13 |
| KEGG_BASAL_CELL_CARCINOMA | 9.89E-13 |
| KEGG_PRION_DISEASES | 3.39E-11 |
| KEGG_ECM_RECEPTOR_INTERACTION | 6.48E-11 |
| KEGG_FRUCTOSE_AND_MANNOSE_METABOLISM | 9.79E-11 |
| KEGG_SYSTEMIC_LUPUS_ERYTHEMATOSUS | 1.23E-10 |
| KEGG_STARCH_AND_SUCROSE_METABOLISM | 1.97E-10 |
| KEGG_THYROID_CANCER | 4.58E-10 |
| KEGG_CARDIAC_MUSCLE_CONTRACTION | 4.80E-10 |
| KEGG_PRIMARY_IMMUNODEFICIENCY | 7.99E-10 |
| KEGG_COLORECTAL_CANCER | 8.03E-10 |
| KEGG_RIBOFLAVIN_METABOLISM | 1.68E-09 |
| KEGG_CALCIUM_SIGNALING_PATHWAY | 1.84E-09 |
| KEGG_ETHER_LIPID_METABOLISM | 2.36E-09 |
| KEGG_TIGHT_JUNCTION | 8.32E-09 |
| KEGG_PORPHYRIN_AND_CHLOROPHYLL_METABOLISM | 1.31E-08 |
| KEGG_ARACHIDONIC_ACID_METABOLISM | 2.22E-08 |
| KEGG_CYTOSOLIC_DNA_SENSING_PATHWAY | 3.82E-08 |
| KEGG_ARRHYTHMOGENIC_RIGHT_VENTRICULAR_CARDIOMYOPATHY_ARVC | 9.40E-08 |
| KEGG_AMINO_SUGAR_AND_NUCLEOTIDE_SUGAR_METABOLISM | 1.82E-07 |
| KEGG_NEUROTROPHIN_SIGNALING_PATHWAY | 2.39E-07 |
| KEGG_PATHOGENIC_ESCHERICHIA_COLI_INFECTION | 2.39E-07 |
| KEGG_PROTEASOME | 2.77E-07 |
| KEGG_VIRAL_MYOCARDITIS | 3.31E-07 |
| KEGG_CIRCADIAN_RHYTHM_MAMMAL | 4.27E-07 |
| KEGG_P53_SIGNALING_PATHWAY | 4.41E-07 |
| KEGG_INTESTINAL_IMMUNE_NETWORK_FOR_IGA_PRODUCTION | 4.52E-07 |
| KEGG_NITROGEN_METABOLISM | 5.32E-07 |
| KEGG_GLYCOSPHINGOLIPID_BIOSYNTHESIS_GANGLIO_SERIES | 5.32E-07 |
| KEGG_N_GLYCAN_BIOSYNTHESIS | 7.56E-07 |
| KEGG_TYPE_II_DIABETES_MELLITUS | 9.33E-07 |
| KEGG_GLYCEROLIPID_METABOLISM | 9.93E-07 |
| KEGG_CHRONIC_MYELOID_LEUKEMIA | 2.15E-06 |
| KEGG_SNARE_INTERACTIONS_IN_VESICULAR_TRANSPORT | 2.15E-06 |
| KEGG_PROGESTERONE_MEDIATED_OOCYTE_MATURATION | 2.25E-06 |
| KEGG_SULFUR_METABOLISM | 2.28E-06 |
| KEGG_DORSO_VENTRAL_AXIS_FORMATION | 5.94E-06 |
| KEGG_OTHER_GLYCAN_DEGRADATION | 6.27E-06 |
| KEGG_HEDGEHOG_SIGNALING_PATHWAY | 1.76E-05 |
| KEGG_PRIMARY_BILE_ACID_BIOSYNTHESIS | 1.91E-05 |
| KEGG_NATURAL_KILLER_CELL_MEDIATED_CYTOTOXICITY | 2.08E-05 |
| KEGG_LINOLEIC_ACID_METABOLISM | 2.82E-05 |
| KEGG_GLIOMA | 3.34E-05 |
| KEGG_AUTOIMMUNE_THYROID_DISEASE | 4.92E-05 |
| KEGG_BASAL_TRANSCRIPTION_FACTORS | 5.06E-05 |
| KEGG_PHOSPHATIDYLINOSITOL_SIGNALING_SYSTEM | 5.55E-05 |
| KEGG_STEROID_BIOSYNTHESIS | 9.90E-05 |
| KEGG_FOLATE_BIOSYNTHESIS | 0.000113 |
| KEGG_LYSOSOME | 0.000115 |
| KEGG_MISMATCH_REPAIR | 0.000118 |
| KEGG_LEUKOCYTE_TRANSENDOTHELIAL_MIGRATION | 0.000135 |
| KEGG_OOCYTE_MEIOSIS | 0.000135 |
| KEGG_NUCLEOTIDE_EXCISION_REPAIR | 0.000146 |
| KEGG_JAK_STAT_SIGNALING_PATHWAY | 0.000165 |
| KEGG_ASTHMA | 0.000193 |
| KEGG_BASE_EXCISION_REPAIR | 0.000275 |
| KEGG_TYPE_I_DIABETES_MELLITUS | 0.000298 |
| KEGG_FC_EPSILON_RI_SIGNALING_PATHWAY | 0.000484 |
| KEGG_RNA_DEGRADATION | 0.000515 |
| KEGG_RETINOL_METABOLISM | 0.000515 |
| KEGG_GRAFT_VERSUS_HOST_DISEASE | 0.000517 |
| KEGG_MELANOMA | 0.000524 |
| KEGG_PENTOSE_PHOSPHATE_PATHWAY | 0.001059 |
| KEGG_MELANOGENESIS | 0.001177 |
| KEGG_GLYCOSPHINGOLIPID_BIOSYNTHESIS_GLOBO_SERIES | 0.001177 |
| KEGG_ALLOGRAFT_REJECTION | 0.001353 |
| KEGG_CHEMOKINE_SIGNALING_PATHWAY | 0.00141 |
| KEGG_ALDOSTERONE_REGULATED_SODIUM_REABSORPTION | 0.001538 |
| KEGG_PROTEIN_EXPORT | 0.001983 |
| KEGG_GLYCOSAMINOGLYCAN_BIOSYNTHESIS_HEPARAN_SULFATE | 0.002354 |
| KEGG_NOTCH_SIGNALING_PATHWAY | 0.002699 |
| KEGG_FC_GAMMA_R_MEDIATED_PHAGOCYTOSIS | 0.003284 |
| KEGG_ALPHA_LINOLENIC_ACID_METABOLISM | 0.003597 |
| KEGG_VASCULAR_SMOOTH_MUSCLE_CONTRACTION | 0.003986 |
| KEGG_SMALL_CELL_LUNG_CANCER | 0.004019 |
| KEGG_AXON_GUIDANCE | 0.004817 |
| KEGG_REGULATION_OF_ACTIN_CYTOSKELETON | 0.005754 |
| KEGG_NOD_LIKE_RECEPTOR_SIGNALING_PATHWAY | 0.006255 |
| KEGG_CELL_ADHESION_MOLECULES_CAMS | 0.007331 |
| KEGG_O_GLYCAN_BIOSYNTHESIS | 0.007787 |
| KEGG_LEISHMANIA_INFECTION | 0.014038 |
| KEGG_FOCAL_ADHESION | 0.015183 |
| KEGG_ABC_TRANSPORTERS | 0.016203 |
| KEGG_APOPTOSIS | 0.017091 |
| KEGG_MAPK_SIGNALING_PATHWAY | 0.019137 |
| KEGG_ANTIGEN_PROCESSING_AND_PRESENTATION | 0.022802 |
| KEGG_GALACTOSE_METABOLISM | 0.02497 |
| KEGG_HOMOLOGOUS_RECOMBINATION | 0.035186 |
| KEGG_OXIDATIVE_PHOSPHORYLATION | 0.043701 |
| KEGG_GNRH_SIGNALING_PATHWAY | 0.051573 |
| KEGG_CELL_CYCLE | 0.060773 |
| KEGG_GLYCOSPHINGOLIPID_BIOSYNTHESIS_LACTO_AND_NEOLACTO_SERIES | 0.062106 |
| KEGG_LONG_TERM_POTENTIATION | 0.076233 |
| KEGG_DRUG_METABOLISM_CYTOCHROME_P450 | 0.078607 |
| KEGG_VEGF_SIGNALING_PATHWAY | 0.080406 |
| KEGG_PURINE_METABOLISM | 0.080542 |
| KEGG_RIG_I_LIKE_RECEPTOR_SIGNALING_PATHWAY | 0.080549 |
| KEGG_HUNTINGTONS_DISEASE | 0.086122 |
| KEGG_PARKINSONS_DISEASE | 0.113805 |
| KEGG_TYROSINE_METABOLISM | 0.135135 |
| KEGG_TGF_BETA_SIGNALING_PATHWAY | 0.151933 |
| KEGG_LONG_TERM_DEPRESSION | 0.186203 |
| KEGG_RNA_POLYMERASE | 0.189511 |
| KEGG_WNT_SIGNALING_PATHWAY | 0.205528 |
| KEGG_ALZHEIMERS_DISEASE | 0.209384 |
| KEGG_MATURITY_ONSET_DIABETES_OF_THE_YOUNG | 0.218471 |
| KEGG_PHENYLALANINE_METABOLISM | 0.227562 |
| KEGG_BLADDER_CANCER | 0.250835 |
| KEGG_GLUTATHIONE_METABOLISM | 0.290213 |
| KEGG_GLYCOSAMINOGLYCAN_DEGRADATION | 0.332703 |
| KEGG_GLYCEROPHOSPHOLIPID_METABOLISM | 0.344761 |
| KEGG_SPLICEOSOME | 0.361206 |
| KEGG_TOLL_LIKE_RECEPTOR_SIGNALING_PATHWAY | 0.361206 |
| KEGG_STEROID_HORMONE_BIOSYNTHESIS | 0.467238 |
| KEGG_PYRIMIDINE_METABOLISM | 0.540292 |
| KEGG_GAP_JUNCTION | 0.542591 |
| KEGG_METABOLISM_OF_XENOBIOTICS_BY_CYTOCHROME_P450 | 0.662485 |
| KEGG_T_CELL_RECEPTOR_SIGNALING_PATHWAY | 0.743619 |
| KEGG_ACUTE_MYELOID_LEUKEMIA | 0.749139 |
| KEGG_B_CELL_RECEPTOR_SIGNALING_PATHWAY | 0.783485 |
| KEGG_DNA_REPLICATION | 0.783485 |
| KEGG_DRUG_METABOLISM_OTHER_ENZYMES | 0.795049 |
| KEGG_PATHWAYS_IN_CANCER | 0.880664 |
| KEGG_VIBRIO_CHOLERAE_INFECTION | 0.946726 |
