# Supplementary material for: The core genes of cuproptosis assists in discerning prognostic and immunological traits of clear cell renal cell carcinoma
Source: Front Oncol. 2022 Sep 21;12:925411. doi: 10.3389/fonc.2022.925411 (PMC9533068; doi:10.3389/fonc.2022.925411)
Supplement: Supplementary file 7 [file Table_3.docx]

**Table S3.** Correlation between expression of fdx1 and clinicopathological features

| Clinical features | FDX1 expression | | *P* |
| --- | --- | --- | --- |
|  | High(n) | Low(n) |  |
| Age |  |  | 0.5648 |
| <65 | 42 | 36 |  |
| ≥65 | 23 | 36 |  |
| Gender |  |  | 0.862 |
| Male | 41 | 41 |  |
| Female | 24 | 21 |  |
| T stage |  |  | 0.1776 |
| T1 | 62 | 53 |  |
| T2 | 2 | 5 |  |
| T3 | 0 | 3 |  |
| T4 | 1 | 1 |  |
| N stage |  |  | 0.957 |
| N0 | 63 | 59 |  |
| N1 | 2 | 3 |  |
| M stage |  |  | 0.2262 |
| M0 | 65 | 59 |  |
| M1 | 0 | 3 |  |
| Grade |  |  | 0.0005 |
| G1 | 27 | 10 |  |
| G2 | 30 | 26 |  |
| G3 | 5 | 18 |  |
| G4 | 3 | 8 |  |
